# Effect of Mud Powder on the Performance of Bridge Deck Leveling Concrete in a Seasonally Frozen Region

**DOI:** 10.3390/ma16206793

**Published:** 2023-10-20

**Authors:** Chenglin Shi, Ruize Zhao, Wensheng Wang

**Affiliations:** 1 College of Transportation Science and Engineering, Jilin Jianzhu University, Changchun 130118, China; 2 Kay Laboratory of Architectural Cold Climate Energy Management, Ministry of Education, Changchun 130118, China; 3 College of Transportation, Jilin University, Changchun 130022, China

**Keywords:** mud powder, mechanical properties, durability, seasonally frozen region, bridge deck leveling concrete, interfacial transition zones, microhardness

## Abstract

Mud powders in aggregates are often found to cause deterioration of concrete properties. Based on a study of the mechanical properties of bridge deck leveling concrete (BDLC) containing different mud powders at various ages, the effects of mud powders on concrete durability were evaluated through rapid chloride permeability testing, freeze–thaw testing, and the coupling of salt solution and a freeze–thaw test. The properties of the interfacial transition zone (ITZ) were also investigated via microhardness testing. The test results showed that mud powder reduced the compressive strength, static compressive elastic modulus, and bond strength at early stages of curing. Moreover, mud powder was found to reduce the tensile properties and durability of concrete, with clay powder causing a greater reduction than mud powder in river sands and coarse aggregate. In addition, the width of the ITZ of concrete containing mud powder was found to increase by 23.1–48.3%. A significant correlation between the ITZ and the tensile properties, as well as the durability of concrete, was also observed. Therefore, in order to improve the tensile properties and durability of BDLC in seasonally frozen regions, the content of mud powder in the aggregates should be minimized according to the different compositions of mud powders. The coupling effect of salt solution and a freeze–thaw cycle should also be taken into consideration.

## 1. Introduction

In recent years, increasingly heavy axle loads and surging traffic volumes have led to the occurrence of cracks, pumping distress, interlayer bond failures, and other issues in local areas of bridge decks. This is mainly attributed to the early deterioration of the bridge deck pavement layer, allowing water ingress into the bridge structure [1,2,3,4]. Simultaneously, the bridge experiences dynamic stress factors, such as temperature fluctuations and the impact of passing vehicles. These stressors lead to the expansion and contraction of the bridge girders, rotation at the girders’ ends, and vertical misalignment between adjacent bridge sections, due to the unsynchronized compression of rubber bearings, resulting in structural deformities and damage to the bridge deck’s pavement layer [5,6]. As an important component of the bridge superstructure, the leveling layer supports the bridge deck asphalt pavement layer and connects to the bridge girders. It plays dual roles of top and bottom, serving to adjust and control elevation, transfer loads, and provide waterproofing and anti-corrosion protection [7]. The leveling layer is crucial, functioning to both bear the pavement layer above and connect to the girders below, facilitating elevation adjustment while transferring loads and preventing water and corrosion ingress.

The strength of concrete stands as the foremost factor in structural design; however, in numerous practical scenarios, durability assumes primacy [8,9]. Within seasonally frozen regions, the recurring freeze–thaw cycles can result in the deterioration of both the mechanical properties and durability of concrete [10,11,12]. Of more significant concern is the post-winter snowfall application of de-icing salt onto the bridge deck. The ensuing ice–snow melting water, replete with de-icing salt, infiltrates the bridge deck leveling concrete (BDLC) through imperfections in the paving layer [2,13]. The combined effect of the salt solution and the freeze–thaw cycles expedites the degradation of the concrete structure [14,15,16,17]. Simultaneously, chloride ions within the saline solution exhibit the capacity to degrade the oxidized layer encompassing steel reinforcement within the BDLC [18]. This action serves to exacerbate the corrosion of the steel reinforcement [19,20], resulting in a substantial reduction in the overall durability of the BDLC [21,22]. This, in turn, can potentially lead to the erosion of the bridge girder, posing a significant threat to the structural integrity of the bridge. Consequently, ensuring both the service safety and the durability of bridges within seasonally frozen regions hinges on the paramount significance of employing high-quality BDLC materials in bridge construction.

China defines the content of fine particles in aggregates as the proportion of particles with a particle size less than 75 μm [23,24]. In practical construction projects, concrete aggregates inevitably contain these fine particles. The fine particles in aggregates have the capacity to adsorb both water reducers and water itself [25,26]. It is important to note that different compositions of fine particles exhibit variations in their adsorption capabilities for water reducers and water [27]. Concrete comprises three distinct phases: aggregates, cement paste, and the interfacial transition zone (ITZ). Often, the ITZ represents a critical factor in the context of concrete properties, serving as a potential “weak link” [28,29]. Moreover, a clear correlation exists between the characteristics and properties of the ITZ and the mechanical properties and durability of concrete [30,31,32].

As a portion of the fine mud powder resides on the surface of aggregates in the form of a surface cover layer, it obstructs the bonding between aggregates and the cementitious paste, potentially leading to a reduction in both the strength and durability of the concrete [33]. Studies by Nehdi [34] and Hamed et al. [35] have revealed that nano-clay can enhance the mechanical properties of concrete by positively influencing the microstructure of cementitious materials. Similarly, Ashraf et al. [36] have demonstrated that the combination of silica fume and bentonite clay, as supplementary cementitious materials, can result in high-strength concrete, particularly when subjected to extended curing periods. However, to date, a comprehensive and systematic elucidation of the impact of various types of mud powders on the properties of the ITZ in concrete remains elusive. Moreover, limited comparative studies have been undertaken to investigate the effects of different mud powders found in river sands and coarse aggregates, in conjunction with clay powder, on the overall properties of concrete.

In this study, taking into account the operational requirements of BDLC in seasonally frozen regions, we conducted a comprehensive comparative analysis to assess the influence of various compositions of mud powder on the mechanical properties of BDLC. Our evaluation encompassed the relative permeability of different concrete mixtures, employing the electrical flux method. Additionally, we designed freeze–thaw tests, as well as the coupling of salt solution and freeze–thaw tests, to gauge the frost resistance of concrete. Parameters such as mass loss rate, relative dynamic elastic modulus, and relative durability index were considered. The characteristics of the ITZ were examined at a microscopic scale through the incorporation of microhardness testing. We also conducted a comprehensive assessment of how various types of mud powder influence the performance of BDLC. The primary objective of this research is to conduct a comparative and analytical study of the impacts of commonly encountered mud powder varieties on the mechanical properties and durability of concrete in practical engineering applications. The ultimate aim is to establish a foundational understanding for the construction of BDLC in regions characterized by seasonal freezing conditions.

## 2. Materials and Methods

### 2.1. Materials and Mix Designs

Based on the construction technical plan for the Jiamusi Transit Section PPP Project of the HeDa Expressway, specifically for the Songhua River Bridge, the primary cementitious materials employed were ordinary Portland cement with a strength grade of 52.5 [37] and fly ash, classified as class Ⅰ [38]. Detailed information regarding the chemical compositions and physical characteristics of these materials can be found in Table 1. The primary constituents for this construction included coarse aggregates and river sands, and their essential properties are detailed in Table 2. The principal characteristics of the polypropylene fiber are detailed in Table 3. To further improve the workability of the concrete, a polycarboxylate superplasticizer (RAWY-101) produced by Anhui Zhongtie Engineering Material Science and Technology Co., Ltd. (Hefei, China) was employed. This superplasticizer facilitated the achievement of a consistent slump of 150 ± 10 mm in the fresh concrete. Additionally, an inhibitor (SBT-TIA) from Jiangsu Subote New Material Co., Ltd. (Nanjing, China) was incorporated to enhance the frost resistance of the concrete.

**Figure 1:**
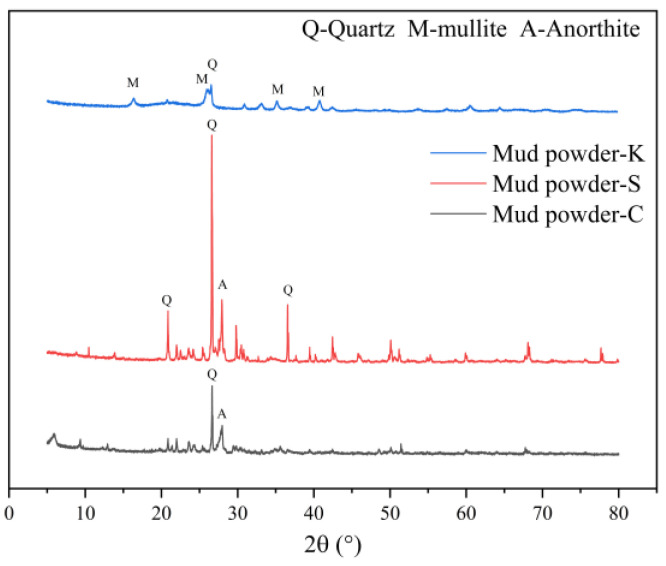
XRD patterns of mud powder, Mud powder-C, Mud powder-S and Mud powder-K are obtained by sieving from unwashed coarse aggregate and unwashed river sands, with particles sizes below 75 μm, and clay powder primarily composed of kaolin.

The river sands and coarse aggregates underwent a cleaning process using water. After this cleaning, the resulting mud liquid, which included materials from both the river sands and coarse aggregates, was passed through a square-hole sieve with a mesh size of 75 μm. This step ensured that particles with a size exceeding 75 μm within the aggregates were retained and not washed away. To facilitate a meaningful comparison of the effects of various mud powder compositions on concrete performance, the mud content of the aggregates utilized in the real-world project was adopted as the mud content benchmark for the test mixture. Furthermore, to maintain control over the variables, different mud powders were introduced into the cleaned aggregates, according to the specifications outlined in Table 4. The chemical composition of the mud powder was ascertained using an X-ray fluorescence spectrometer (Zetium, PANalytical B.V., Beijing, China). An X-ray diffractometer (Ultima IV, Shanghai Sibaiji Instrument System Co., Ltd., Shanghai, China) was employed to analyze the presence of various mineral phases within the mud powder. The test results, which include the chemical composition data and the mineral phase analysis, are presented in Table 5 and Figure 1, respectively. To determine the most suitable mixture proportions, preliminary experiments were conducted for different combinations. Subsequently, the final compositions for BDLC mixes were established, and these are detailed in Table 6.

### 2.2. Specimen Preparation and Curing

The concrete mixtures were prepared using a double horizontal shaft laboratory mixer. Once mixed, the specimens were compacted into molds and left to demold for 1 d at a 20 ± 5 °C and relative humidity of more than 50%. Following the demolding stage, the specimens were subjected to standard curing conditions, with humidity levels nominally at 95% and a temperature of approximately 23 °C, until they were ready for testing.

### 2.3. Mechanical Properties Tests

**Figure 2:**
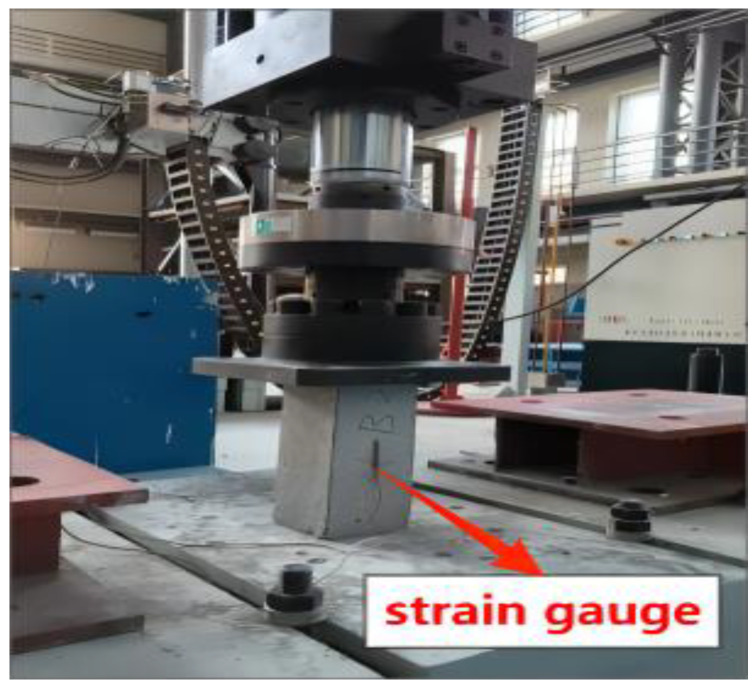
Static compressive elastic modulus test.

In accordance with the guidelines outlined in JTG3420-2020 [39], a series of tests were carried out to assess various properties of the concrete. These tests encompassed concrete compressive strength, split tensile strength, static compressive elastic modulus, and flexural-tensile strength. The compressive strength test and static compressive elastic modulus test were executed with a loading rate of 0.5 MPa/s, while the splitting tensile strength and flexural-tensile strength tests were performed with a loading rate of 0.05 MPa/s. Notably, for the static compressive elastic modulus test, a multidimensional MAS-500 servo press (Hangzhou Bangwei Electromechanical Control Engineering Co., Ltd., Hangzhou, China) was employed, due to its requisite sensitivity and stability, as depicted in Figure 2.

**Figure 3:**
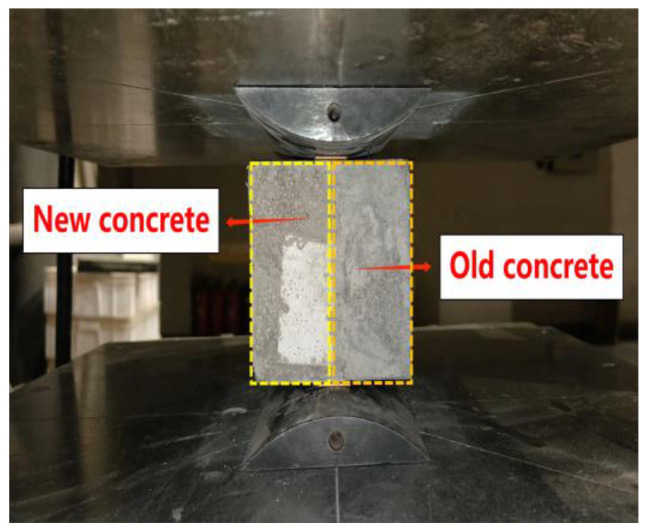
Splitting tensile bond strength test.

A time gap exists between the casting of the BDLC and the girder concrete. As a result, a test was designed to assess the splitting tensile bond strength, with reference to the Chinese specification GB/T50081-2019 [40], as depicted in Figure 3. In this test, the existing concrete employs identical raw materials and proportions as the new concrete it will be bonded to. The concrete surface was intentionally roughened using a manual chiseling method, and then the specimens were cured under standard conditions for 56 d. Given that surface roughness can significantly influence bond strength [41,42,43,44], a method for assessing roughness was employed using the sand filling technique. This process involved placing a plastic plate around the boundary of the bonding surface of the specimen. The upper surface of the plastic plate was aligned with the highest point of the raised portion on the bonding surface. Fine standard sand was then introduced between the bonding surface and the upper surface of the plastic plate. To quantitatively describe the roughness of the bonding surface, calculations were made using Equation (1):

(1) H=V/A

where *H* represents the roughness (mm); *V* denotes the standard sand volume (mL); *A* is the cross-sectional area of the specimen (${\mathrm{mm}}^{2}$).

In order to provide a more precise evaluation of the bonding characteristics between the old and new concrete, specimens with a predefined roughness level of 3.6 ± 0.3 were carefully selected. The testing procedure involved immersing these specimens in water for a duration of 6 h. After this immersion, the specimens were taken out, covered with damp cloths, cleaned after a further 12 h, and subsequently allowed to dry until no visible water remained on their surfaces. Following this preparation, concrete was poured to conduct the tests. For the splitting tensile bond strength assessment, a loading rate of 0.25 kN/s was applied. Each group comprised 6 specimens, and the highest and lowest values obtained from the test results were discarded. The remaining 4 values were then averaged to yield the final test results.

### 2.4. Rapid Chloride Permeability Test

Chloride salt is recognized as the predominant component of de-icing salt in regions subject to seasonal freezing [13]. Consequently, the rapid chloride permeability test was conducted to evaluate concrete specimens, using electrical flux measurements as an indicator. This test adhered to the standards set forth in JTG3420-2020 [39] and ASTMC1202 [45]. The testing procedure involved initially exposing the specimens to air until the surface was completely dry. Subsequently, the cylindrical sides of the specimens were coated with silica gel. A Model NEL-VJH fully automated vacuum water saturation machine (Beijing Nair intelligent Technology Co., Ltd., Beijing, China) was utilized to achieve vacuum water saturation of the sealed specimens. The saturated specimens were then placed within a test tank, and the seal between the specimens and the test tank was verified using distilled water. Subsequently, the copper mesh in the test tank containing a 3.0% NaCl solution with a mass concentration was connected to the negative pole of the power supply. Simultaneously, the copper mesh in the test tank containing a 0.3 mol/L NaOH solution with a molar concentration was connected to the positive pole of the power supply. A constant direct current (DC) voltage of 60 volts was then applied to the copper mesh for a duration of 6 h. During this 6-h period, readings were taken at 15-min intervals using a device designed for automated data collection. At the conclusion of the 6-h period, the electrical flux through the specimens was calculated based on the collected data.

### 2.5. Freeze–Thaw Test

**Figure 4:**
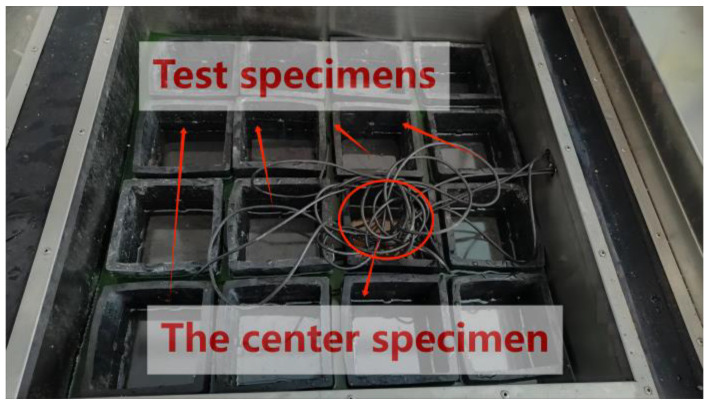
Placement of the specimens for freeze–thaw test.

**Figure 5:**
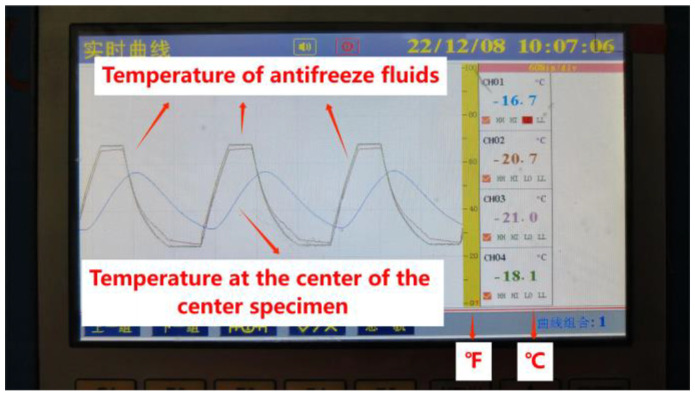
Temperature variation for freeze-thaw test.

The freeze–thaw test was conducted using the quick-freeze method in accordance with the guidelines outlined in JTG3420-2020 [39]. Specimens that had been cured for up to 24 d were submerged in water for a period of 4 d. After this immersion, the surface moisture was wiped off with a damp cloth. Subsequently, the transverse fundamental frequency of the specimens was measured and their weight recorded as the initial values for assessing the freeze–thaw resistance of the concrete. For the freeze–thaw test, the specimens were placed into elastic rubber test tanks filled with water, as illustrated in Figure 4. In the case of the coupling of salt solution and freeze–thaw test, the specimens were instead immersed in rubber test tanks containing a NaCl solution with a mass concentration of 3.0%. Each cycle of freeze–thaw was accomplished over a period of roughly 3 h. During these cycles, the temperature changes within the specimens’ centers, as well as the specific freeze–thaw antifreeze temperature, were documented, as depicted in Figure 5. At intervals of 25 freeze–thaw cycles, the specimens were subjected to testing, which included measuring their weight and transverse fundamental frequency. The test was brought to a conclusion when either of the following two conditions was met:

(a) Mass loss rate exceeding 5%
(b) Relative dynamic modulus of elasticity declining to less than 60%

### 2.6. Microhardness Test

**Figure 6:**
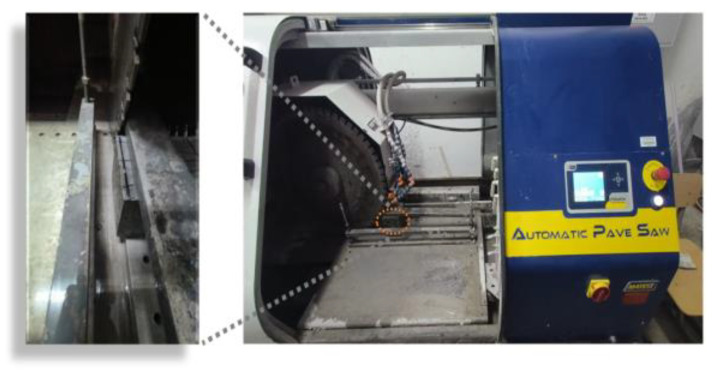
Cutting specimen diagram for microhardness test.

The specimens were sectioned into square slices, each with a thickness of 7 ± 2 mm, utilizing a cutting machine, as illustrated in Figure 6. These slices were further subdivided into smaller segments with the aid of a smaller cutting machine. Subsequently, they were meticulously cut into smaller test pieces, ensuring that the complete aggregates were included, using a metallographic cutter. A sequence of pre-polishing steps was performed, employing 400, 800, and 1200 mesh sandpaper. Following this, a low viscosity epoxy resin was used for vacuum impregnation to fill the pores within the test pieces. Once the epoxy resin had fully cured, anhydrous ethanol was employed as a grinding aid. Successively, 1500, 2000, and 2500 mesh sandpaper were utilized for further polishing. Subsequently, various grades of diamond suspension polishing solutions were employed for a finer polishing process. After the polishing procedure had been successfully carried out, the specimens were placed in an oven at 60 °C for a period of 6 h to facilitate drying.

**Figure 7:**
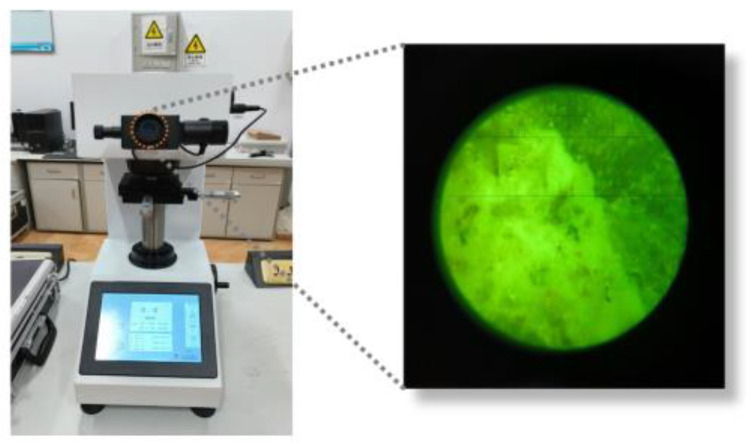
Test diagram for microhardness test.

**Figure 8:**
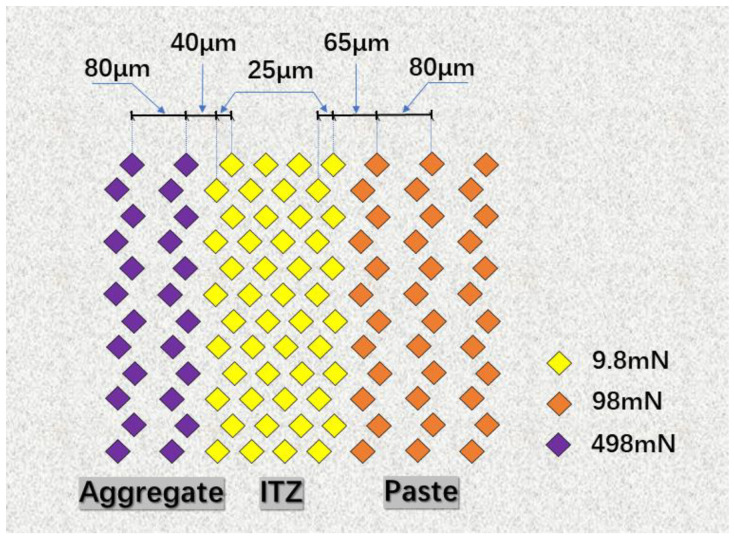
Measurement points of microhardness test.

Vickers hardness testing was employed to assess the properties of the ITZ; images from the eyepiece of the microhardness tester are shown in Figure 7. Initially, the microhardness of the aggregate, paste, and ITZ was pre-tested to obtain preliminary hardness values. Subsequently, an applied load of 9.8 mN was used to assess the ITZ and its surrounding area, while loads of 98 mN and 498 mN were applied to the cement paste and aggregate, respectively. To perform the test, a staggered dot matrix of 9 × 12 was used, with specific measurement points as depicted in Figure 8.

**Figure 9:**
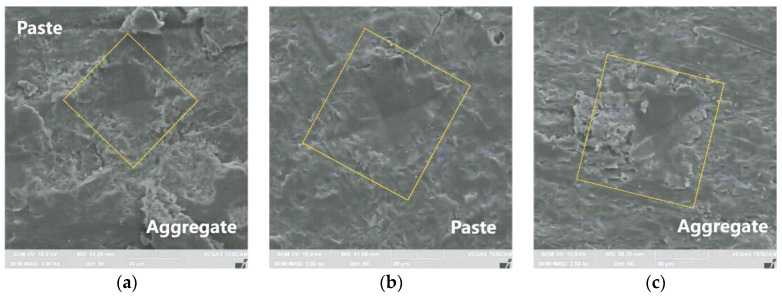
The indents for microhardness test with an applied load of 9.8 mN was used to assess the ITZ, while loads of 98 mN and 498 mN were applied to the paste and aggregate, respectively. (**a**) ITZ, (**b**) Paste, (**c**) Aggregate.

To enhance the precision of measurements, the diagonal length of the indents was determined using a field emission scanning electron microscope (FESEM-TESCAN VEGA3, Tesken Trading (Shanghai) Co., Ltd., Shanghai, China). Subsequently, Vickers hardness values in proximity to the ITZ were computed using Equation (2). The indentations made in the paste, aggregate, and ITZ can be observed in Figure 9a,b and c, respectively.

(2) $$HV=\frac{2P\sin\left(136^{{}^{\circ}}/2\right)}{D^{2}}$$

In which, *HV* is the Vickers hardness (MPa); *P* is the applied load (N); *D* is the average diagonal length of the indents (mm).

## 3. Results and Discussion

### 3.1. Mechanical Properties

#### 3.1.1. Compressive Strength and Static Compressive Elastic Modulus

**Figure 10:**
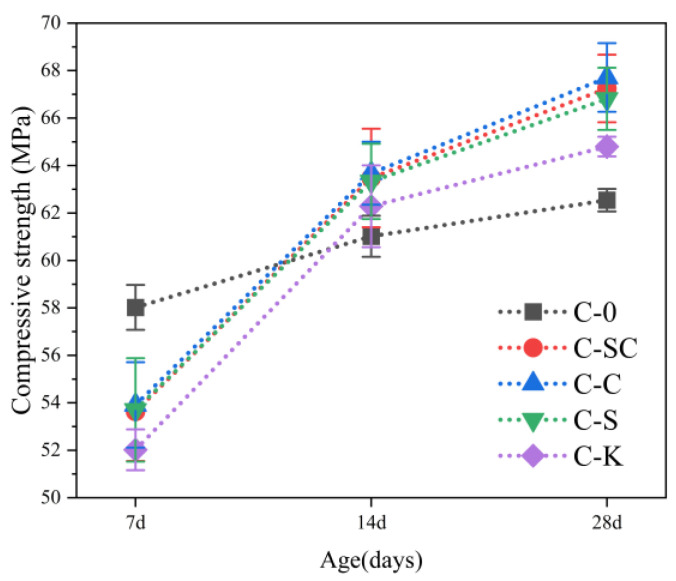
Compressive strength of concrete at various ages.

The test results for compressive strength at various ages are depicted in Figure 10, with error bars indicating standard deviations. Notably, at 7 days, C-0 exhibited the highest compressive strength, surpassing that of C-SC, C-C, C-S, and C-K by 8.2%, 7.6%, 8.0%, and 11.5%, respectively. Surprisingly, at 14 days, the compressive strength of C-SC, C-C, C-S, and C-K had all increased by 18.3%, 18.1%, 17.9%, and 19.7%, respectively and, significantly, they all exceeded the compressive strength of C-0. This phenomenon can be attributed to a dual mechanism. Firstly, the mud powder within the aggregates adsorbs a portion of the water and superplasticizer, thus retarding the cement hydration process, consequently diminishing the early strength of the concrete. Secondly, as the hydration reaction progresses, the mud powder serves to fill the pores within the concrete, enhancing its overall density, and consequently leading to an increase in concrete strength. Moreover, it is evident that, at 28 days, the compressive strength of C-SC, C-C, and C-S surpasses that of C-K. This suggests that clay powder contributes less significantly to the concrete’s strength in comparison to the mud powder found in the river sands and coarse aggregates.

**Figure 11:**
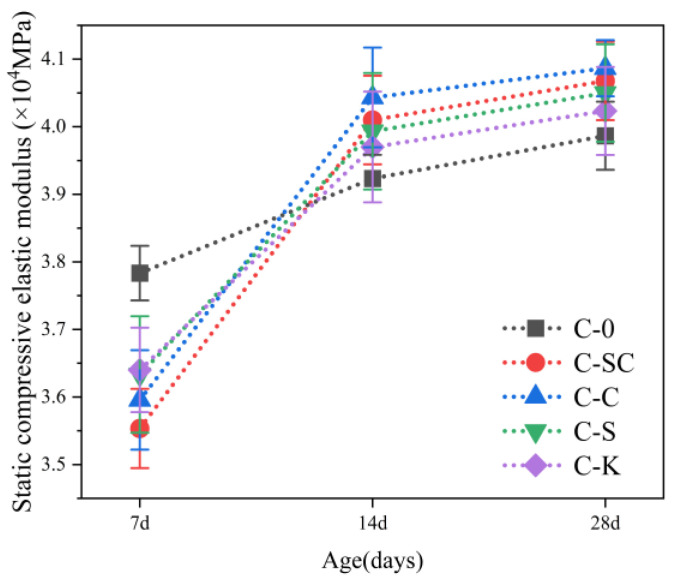
Static compressive elastic modulus of concrete at various ages.

Figure 10 and Figure 11 indicate that the trend observed for the static compressive elastic modulus closely resembles that of compressive strength. However, it is noteworthy that the variation in static compressive elastic modulus between the different groups at the 28-day mark is not particularly significant. This phenomenon might be attributed to the characteristics of the aggregate [46].

**Figure 12:**
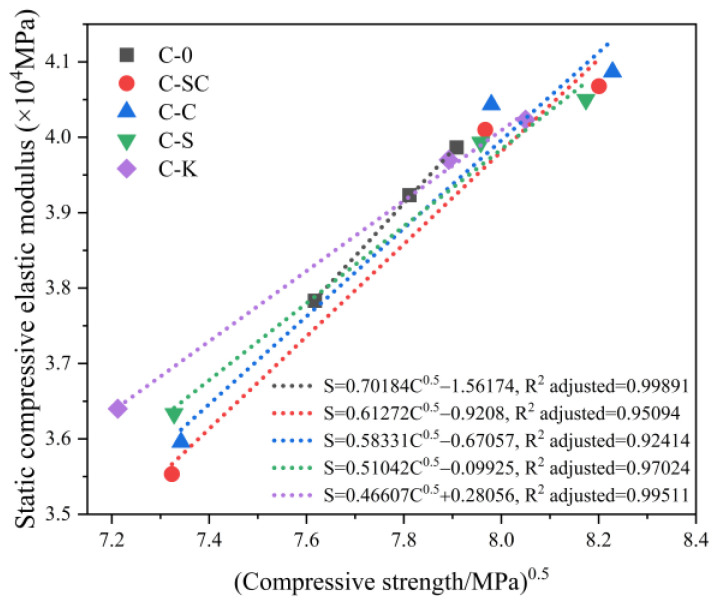
Relationship between compressive strength and static compressive elastic modulus at various ages.

The relationship between the square root of compressive strength and static compressive elastic modulus is illustrated in Figure 12. The data reveal a strong correlation between the square root of compressive strength and the static compressive elastic modulus for C-0, C-SC, C-C, C-S, and C-K. Notably, the R-squared adjusted (R^2^ adjusted) values for C-0 and C-K exceed 0.99, which could be attributed to the uniformity of the mixtures.

#### 3.1.2. Splitting Tensile Strength and Flexural-Tensile Strength

**Figure 13:**
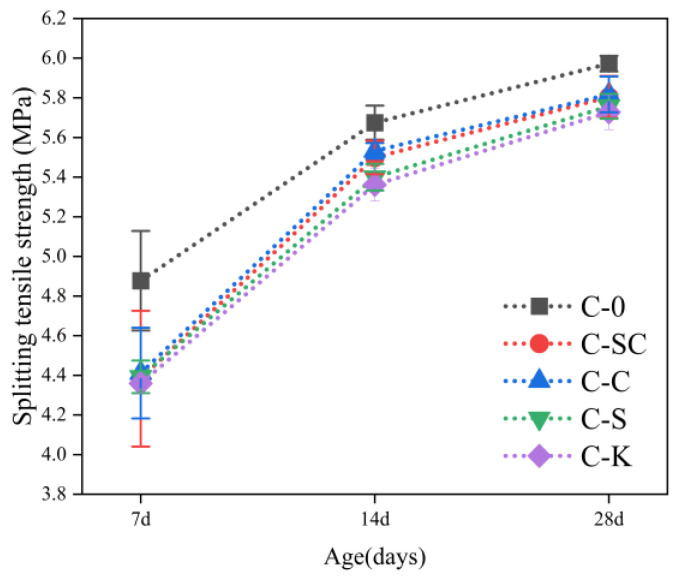
Splitting tensile strength of concrete at various ages.

**Figure 14:**
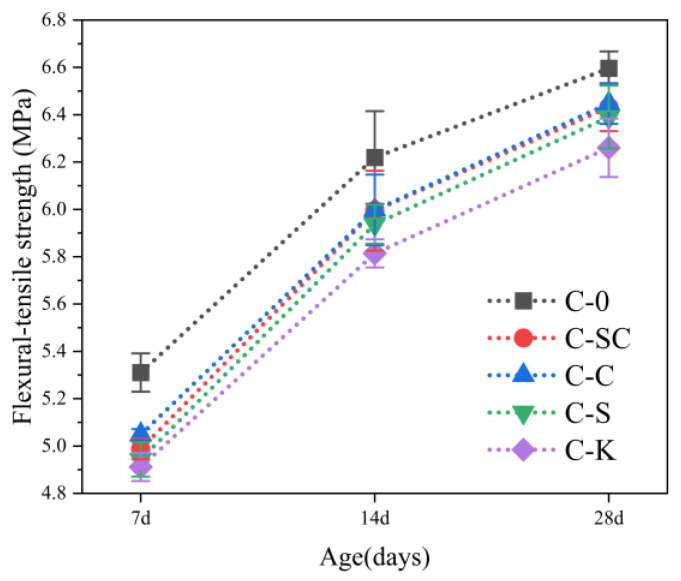
Flexural-tensile strength of concrete at various ages.

The splitting tensile strength of C-0 consistently surpasses that of C-SC, C-C, C-S, and C-K from 7 days to 28 days, as depicted in Figure 13. Specifically, at 7 days, the splitting tensile strength of C-0 is 11.3%, 10.6%, 11.0%, and 11.9% higher than that of C-SC, C-C, C-S, and C-K, respectively. Notably, there is no significant disparity in the splitting tensile strengths of C-SC, C-C, C-S, and C-K. Figure 13 and Figure 14 illustrate that the trend of flexural-tensile strength closely mirrors that of the splitting tensile strength. Additionally, at 28 days, the flexural-tensile strength of C-K is 2.7%, 3.0%, and 2.1% lower than the bending tensile strength of C-SC, C-C, and C-S, respectively.

In summary, C-0 exhibits superior tensile properties, including splitting tensile strength and flexural-tensile strength, when compared to C-SC, C-C, C-S, and C-K. This difference can be attributed to the presence of mud powder in C-SC, C-C, C-S, and C-K, which enhances the overall density of the concrete. However, in these mixtures, the absence of pozzolanic reactions results in poor bonding around the mud powder particles, ultimately leading to reduced tensile properties in the concrete. Furthermore, when compared to the mud powder found in the river sands and coarse aggregate in the actual project, the clay powder has an additional detrimental impact, further diminishing the flexural-tensile strength of the concrete.

**Figure 15:**
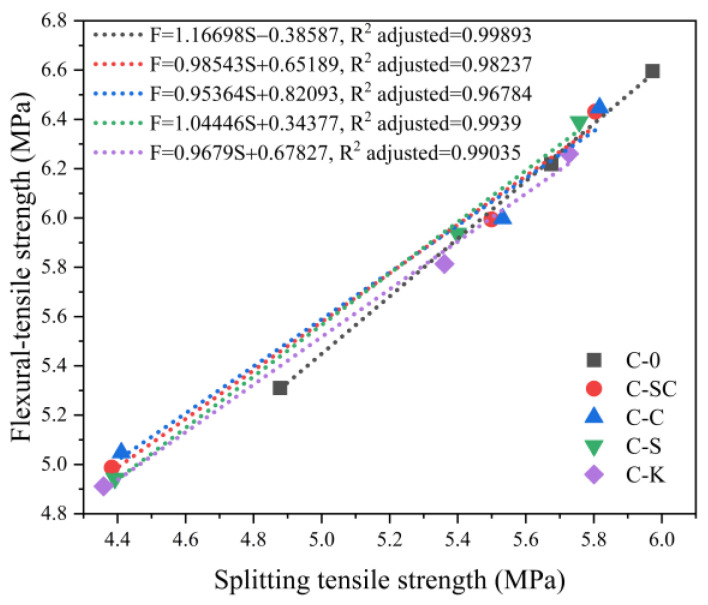
Relationship between splitting tensile strength and flexural-tensile strength at various ages.

The relationship between splitting tensile strength and flexural-tensile strength is illustrated in Figure 15. It is evident that there is a strong correlation between the splitting tensile strength and flexural-tensile strength for C-0, C-SC, C-C, C-S, and C-K, as indicated by the R-squared adjusted values. Furthermore, the slopes of the correlation lines closely approach 1, indicating that the splitting tensile strength and flexural-tensile strength exhibit similar growth rates.

#### 3.1.3. Bond Strength

**Figure 16:**
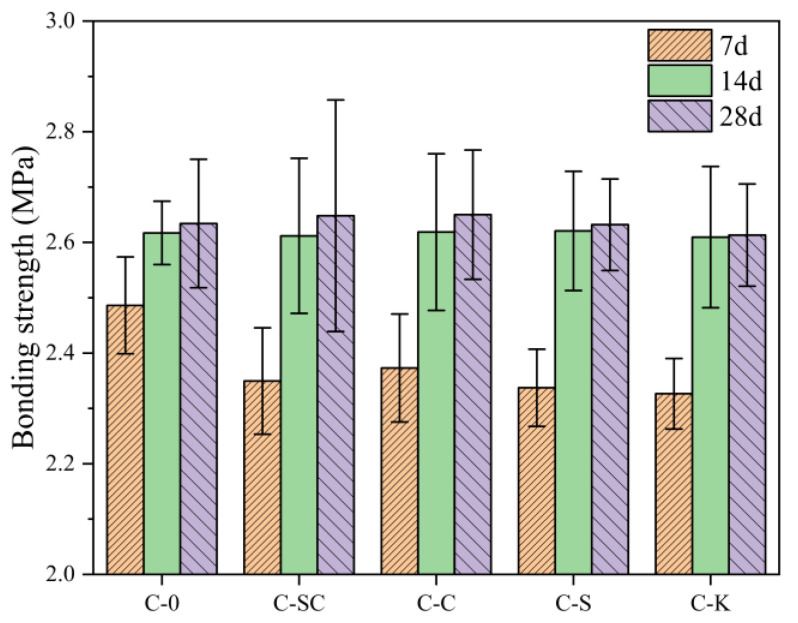
Splitting tensile bond strength of concrete at various ages.

The splitting tensile bond strength at 7 days, 14 days, and 28 days is depicted in Figure 16. At 7 days, the splitting tensile bond strength of C-0 exceeds that of C-SC, C-C, C-S, and C-K by 5.8%, 4.8%, 6.4%, and 6.9%, respectively. However, at 14 days and 28 days, the splitting tensile bond strengths of C-0, C-SC, C-C, C-S, and C-K are nearly identical. This phenomenon can be attributed to two key reasons. Firstly, C-0 undergoes a more extensive degree of early hydration compared to C-SC, C-C, C-S, and C-K, resulting in the presence of relatively fewer defects in the paste at the interface. Another plausible explanation is that the presence of mud powder at the bond surface between the old and new concrete in C-SC, C-C, C-S, and C-K serves to fill the pores created by the large-sized ettringite and calcium hydroxide crystals at the interface. However, it does not significantly contribute to the intergranular bond strength, resulting in relatively lower early bond strength. Notably, the bond strength of C-SC, C-C, C-S, and C-K appear to improve as the hydration reaction progresses. It is also possible that the active silica present in fly ash further reacts with calcium hydroxide at the interface, enhancing the bond strength over time.

In actual construction projects, the compressive strength of BDLC typically meets its performance requirements and has minimal impact on its service life. However, when considering the structure and load-bearing characteristics of BDLC, it becomes evident that the tensile properties hold significantly more importance. While mud powder can enhance the compressive strength of concrete in later stages, it tends to diminish the tensile properties of BDLC, consequently potentially reducing its overall service life.

### 3.2. Durability

#### 3.2.1. Chloride Ion Penetration Resistance

**Figure 17:**
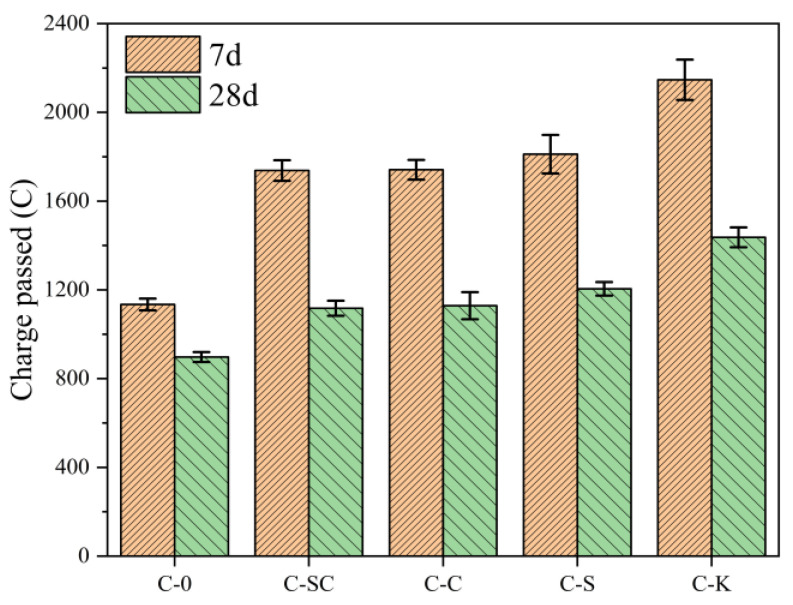
Results of rapid chloride permeability test.

Electrical flux serves as a valuable comparative index for assessing the chloride ion penetration resistance of concretes with different mud powders. The results of the rapid chloride permeability test conducted at 7 days and 28 days are presented in Figure 17. At 7 days, the electrical flux values for C-SC, C-C, C-S, and C-K exceed those of C-0 by 53.2%, 53.5%, 59.7%, and 89.2%, respectively. At 28 days, the electrical flux values for C-0, C-SC, C-C, C-S, and C-K decrease by 26.4%, 55.6%, 54.3%, 50.3%, and 49.4%, respectively. Despite this reduction, the electrical flux values for C-SC, C-C, C-S, and C-K are still notably higher than that of C-0, by 24.5%, 25.8%, 34.3%, and 60.1%, respectively. This observation suggests that mud powder has an adverse impact on the chloride ion penetration resistance of early-age concrete. This effect may be attributed to the fact that mud powder is not evenly distributed throughout the concrete, resulting in the formation of more interconnected pores. Additionally, mud powder can potentially increase the chloride diffusion coefficient within the ITZ [47]. However, as hydration progresses, concrete containing mud powder experiences an improvement in its resistance to chloride ion penetration. Moreover, clay powder has a more pronounced negative impact on the chloride ion penetration resistance of concrete compared to the mud powder found in river sands and coarse aggregates.

#### 3.2.2. Frost Resistance

##### Freeze–Thaw Cycle

**Figure 18:**
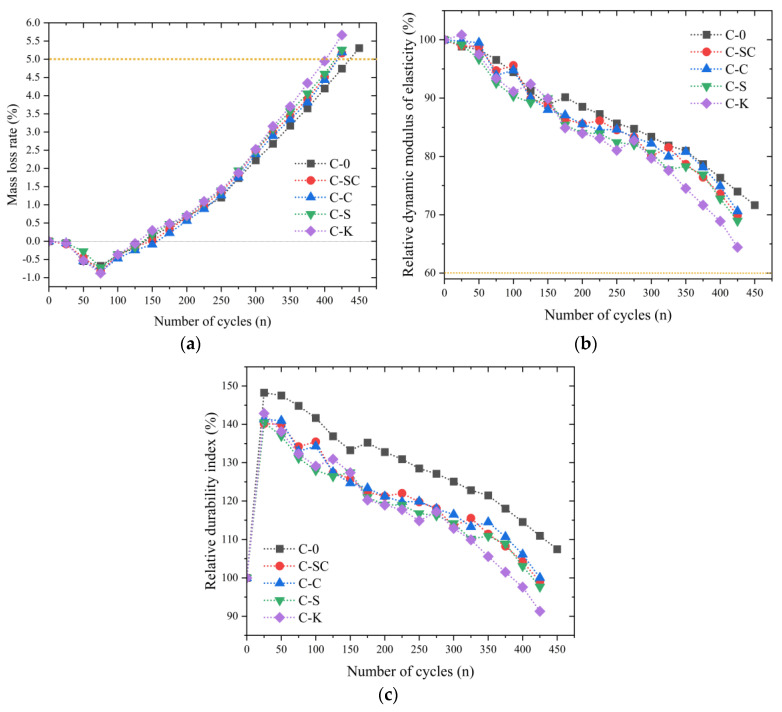
Results of freeze-thaw cycle in water. (**a**) Mass loss rate, (**b**) Relative dynamic modulus of elasticity, (**c**) Relative durability index.

The results of the freeze–thaw cycle in water are presented in Figure 18. In Figure 18a, the mass loss rates of the specimens are depicted for various numbers of cycles. The mass loss rates of each specimen initially exhibit a negative trend during the first 75 cycles. This suggests that the specimens absorb more water mass than they lose due to spalling. However, as the number of cycles continues to increase, the outer layer of the specimens develops more microcracks, leading to an increase in spalling mass. Consequently, this increase in spalling mass contributes to higher mass loss rates for the specimens. This observation aligns with the findings of Pigeon [48]. Moreover, the mass loss rates of each specimen exhibit minimal variation during the initial 300 freeze–thaw cycles. As the testing progresses, C-0 consistently displays lower mass loss rates compared to C-SC, C-C, and C-S. In contrast, the mass loss rates of C-K gradually surpass those of C-SC, C-C, and C-S as the number of freeze–thaw cycles increases. After 425 cycles, the mass loss rates for C-K, C-SC, C-C, and C-S all exceed 5%. Notably, even C-0 exhibits a mass loss rate exceeding 5% after 450 cycles.

The relative dynamic elastic modulus of the specimens exhibits a gradual decline as the freeze–thaw cycles progress. The relative dynamic elastic modulus of C-K significantly lags behind that of C-0, C-SC, C-C, and C-S after 325 freeze–thaw cycles, as illustrated in Figure 18b. As shown in Figure 18a,b, the relative dynamic elastic modulus of C-0, C-SC, C-C, C-S, and C-K remains above 60%, even when the mass loss rates of C-0, C-SC, C-C, C-S, and C-K exceed 5%. This suggests that the freezing zones within the specimens are primarily confined to the outer water-saturated layer at the conclusion of the test. Additionally, it indicates that microcracks have not advanced towards the interior of the specimen, but rather have a tendency to rapidly progress inwards.

Figure 18c shows that, due to its deterioration after 450 freeze–thaw cycles, C-0 exhibits a considerably higher relative durability index when compared to C-SC, C-C, C-S, and C-K. Additionally, the relative durability index of C-0 remains notably higher, surpassing that of C-SC, C-C, C-S, and C-K by 8.6%, 7.5%, 10.1%, and 17.8%, respectively, at the conclusion of the test.

The mass loss rate, relative dynamic elastic modulus, and relative durability index at the conclusion of the test are compiled in Table 7, showing that C-0 withstands a maximum of 450 freeze–thaw cycles, whereas C-SC, C-C, C-S, and C-K endure up to 425 cycles. C-K exhibits the highest mass loss rate, along with the lowest relative dynamic elastic modulus and relative durability index. These findings collectively indicate that C-K has the poorest frost resistance among the tested specimens.

##### Coupling of Salt Solution and Freeze–Thaw Cycle

**Figure 19:**
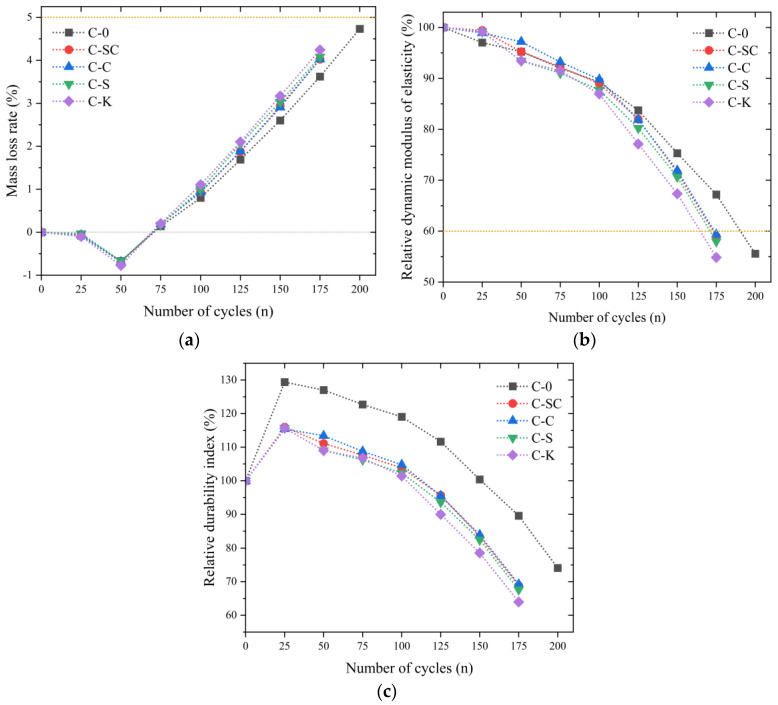
Results of freeze-thaw cycle in salt solution. (**a**) Mass loss rate, (**b**) Relative dynamic modulus of elasticity, (**c**) Relative durability index.

The test results for frost resistance indexes under the combined effect of freeze–thaw and salt solution are presented in Figure 19. Comparing Figure 18a and Figure 19a, it is apparent that the mass loss rate during the coupling of salt solution and freeze–thaw cycles follows a similar trend to that of single freeze–thaw cycles, with the mass of the specimens reaching its maximum after 50 cycles and subsequently gradually decreasing. In the 100–175 cycle range, C-0 exhibits the lowest mass loss rate in comparison to C-SC, C-C, C-S, and C-K. However, after 175 cycles, the mass loss rates of C-SC, C-C, C-S, and C-K surpass that of C-0 by 11.1%, 11.2%, 12.7%, and 17.2%, respectively.

Regarding the relative dynamic elastic modulus, there is minimal distinction between the specimens during the initial 100 cycles, as illustrated in Figure 19b. However, it becomes apparent that C-0 possesses the highest relative dynamic elastic modulus. In the 125–175 cycle range, the relative dynamic elastic modulus of C-K is slightly lower than that of C-SC, C-C, and C-S.

Furthermore, Figure 19a,b and Table 8 show that the mass loss rate does not reach 5% when the relative dynamic elastic modulus falls below 60% at the conclusion of the test. This suggests that the coupling of salt solution and freeze–thaw cycles inflicts a distinct form of damage on the specimens compared to single freeze–thaw cycles. This differentiation may be attributed to the hindrance of supercooled water migration in the gel pores to the ice interface, resulting in the generation of significant osmotic pressure [49]. Consequently, microcracks are formed near the freezing zone of the capillaries in saturated concrete, and the salt solution augments the water saturation of the concrete [50,51], thus accelerating the expansion of the microcracks and causing internal damage to the specimens.

At present, there is no established standard test for evaluating the resistance of concrete to salt freeze–thaw. To provide a more logical assessment of the combined effect of freeze–thaw and salt solution on concrete, the formula for calculating the relative durability index is adjusted from Equation (3) to (4), as per the guidelines outlined in JTG3420-2020 [39]. The results are displayed in Figure 19c. The relative durability index of C-0 surpasses that of C-SC, C-C, C-S, and C-K. The relative durability index of C-SC, C-C, and C-S displays little variation between the 50–175 cycles, while the relative durability index of C-K is marginally lower than that of C-SC, C-C, and C-S.

(3) $$K_{n}=\frac{P\times N}{300}$$

(4) $$K_{n}=\frac{P\times N}{150}$$

where, $K_{n}$ is the relative durability index; *N* is the number of freeze–thaw cycles at which the relative dynamic elastic modulus of the specimen drops below 60% or the mass loss rate of the specimen reaches 5%; *P* is the average value of relative dynamic elastic modulus of three specimens after n freeze–thaw cycles.

In summary, concrete without mud powder exhibits superior frost resistance, while the inclusion of clay powder has a more detrimental effect on frost resistance compared to mud powder present in river sands and coarse aggregate. Moreover, the coupling effect of salt solution and freeze–thaw cycle significantly diminishes the maximum number of freeze–thaw cycles a concrete structure can endure and exacerbates the extent of freeze–thaw damage. To enhance the durability of the BDLC in regions with seasonal freezing conditions, it is advisable to minimize the mud powder content in the aggregates by considering various mud powder compositions and to give strong consideration to the effects of salt solution and freeze–thaw cycles.

### 3.3. Properties of ITZ

**Figure 20:**
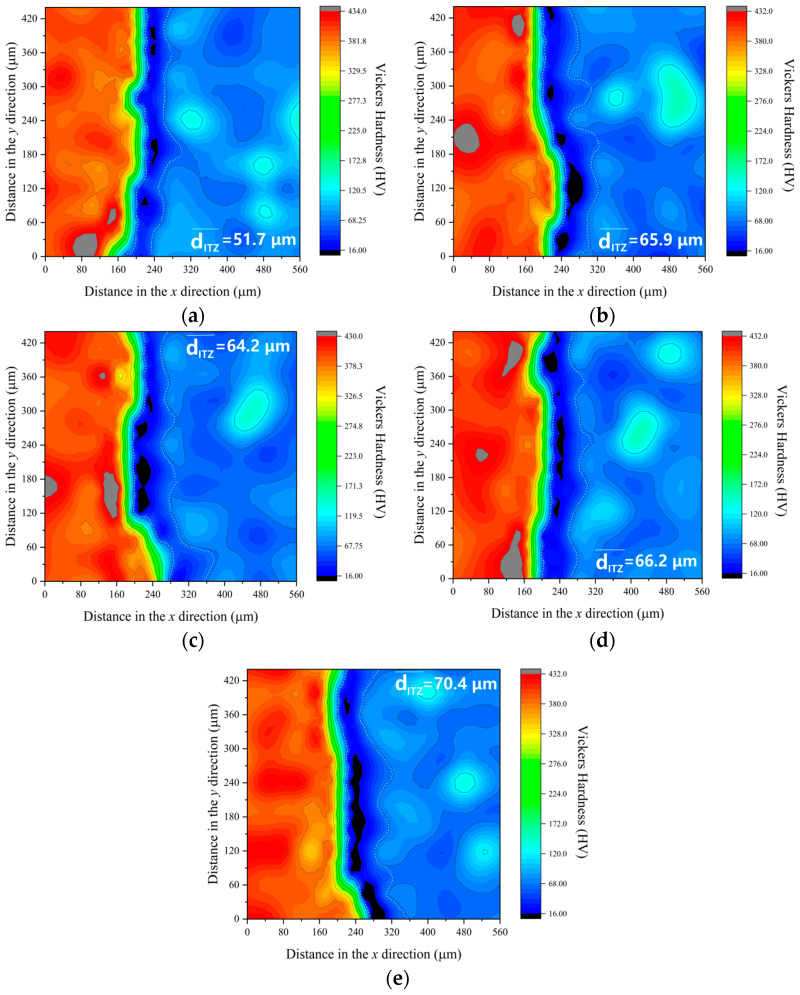
Microhardness of ITZ at 7 days. (**a**) C-0, (**b**) C-SC, (**c**) C-C, (**d**) C-S, (**e**) C-K.

**Figure 21:**
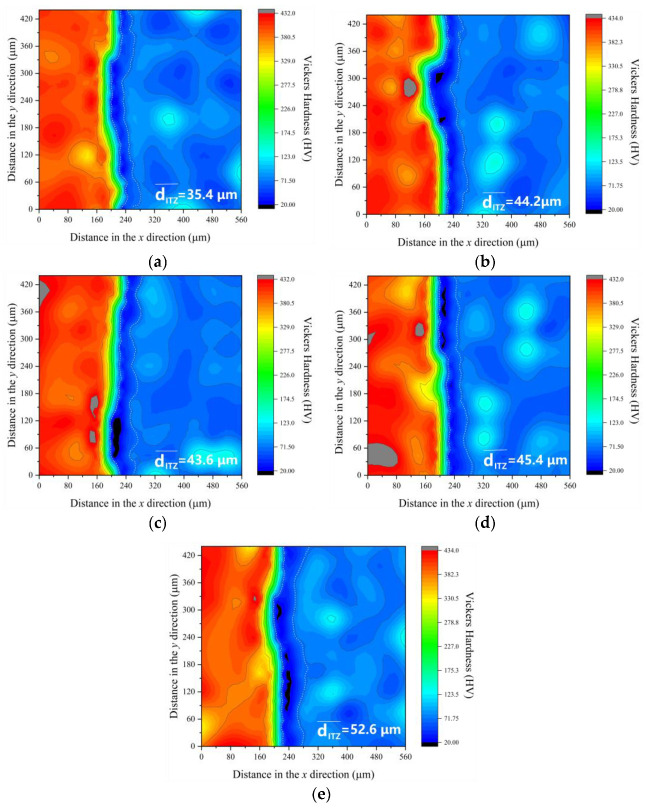
Microhardness of ITZ at 28 days. (**a**) C-0, (**b**) C-SC, (**c**) C-C, (**d**) C-S, (**e**) C-K.

The microhardness data for ITZ at 7 days and 28 days are shown in Figure 20 and Figure 21. The measurement of the ITZ thickness is determined by Equation (5).

(5) $$\bar{d_{ITZ}}=\frac{d_{y_{0}}+d_{y_{60}}+d_{y_{120}}+d_{y_{180}}+d_{y_{240}}+d_{y_{300}}+d_{y_{360}}+d_{y_{420}}}{8}$$

where, $\bar{d_{ITZ}}$ is the thickness of ITZ, μm; $d_{y_{n}}$ is the thickness of the ITZ with vertical coordinate *n*, μm.

At the 7-day mark, C-0 exhibits the thinnest ITZ thickness, with C-SC, C-C, C-S, and C-K showing, respectively, ITZ thicknesses that are 27.7%, 24.3%, 28.1%, and 36.2% greater than C-0. Between 7 and 28 days, the ITZ thickness in C-0 and the other concrete types decreases by 31.4%, 33.0%, 32.1%, 31.4%, and 25.3%, respectively. Even after this reduction, the ITZ thicknesses in C-SC, C-C, C-S, and C-K remain notably higher, with differences of 24.7%, 23.1%, 28.2%, and 48.3%, respectively, in comparison to C-0. This observation underscores the fact that the presence of mud powder reduces the ITZ properties, with clay powder exhibiting the most significant impact in diminishing ITZ thickness.

When considering the results presented in Section 3.1, there is an absence of any discernible correlation between the properties of the ITZ and both compressive strength and static compressive elastic modulus. This observation deviates from the numerical model put forward by Lee [30]. Nonetheless, a noticeable trend emerges: as the ITZ thickness decreases, there is a concurrent increase in tensile properties.

Simultaneously, when we take into account the cumulative impacts of various mud powders on concrete’s chloride ion penetration resistance and frost resistance, as detailed in Section 3.2, it becomes evident that the characteristics of ITZ are intimately connected to concrete’s overall durability.

## 4. Conclusions

The study focused on typical mud powders found in the aggregates employed in a real-world construction project. It conducted a comprehensive comparative analysis of the influence of varying mud powder compositions on concrete’s mechanical attributes and durability. Moreover, the research utilized microhardness measurements to assess the characteristics of the interfacial transition zone (ITZ). The primary findings of this investigation can be summarized as follows:

(1) In the initial stages of curing, concrete without mud powder in aggregates demonstrates superior performance in terms of compressive strength, static compressive elastic modulus, and splitting tensile bond strength. However, the compressive strength and static compressive elastic modulus of the concrete with mud powder surpass those of the concrete without mud powder at the 14 days and 28 days.
(2) During the curing period spanning from 7 days to 28 days, the inclusion of mud powder in the aggregate resulted in a reduction of both the splitting tensile strength and flexural-tensile strength of the concrete. Interestingly, there was a notable positive correlation between the splitting tensile strength and bending tensile strength of the concrete.
(3) Concrete without mud powder demonstrates better resistance to chloride ion penetration, whereas the chloride ion penetration resistance of concrete containing clay powder is lower compared to that of concrete containing mud powder in river sands and coarse aggregate.
(4) The presence of mud powder in aggregates diminishes the early-stage frost resistance of concrete, with clay powder leading to a more substantial reduction in frost resistance compared to mud powder derived from river sands and coarse aggregates. Furthermore, the coupling of salt solution and freeze–thaw cycles significantly intensifies the extent of freeze–thaw damage in comparison to freeze–thaw cycles in water only.
(5) The compressive strength of bridge deck leveling concrete (BDLC) typically fulfills the structural demands for bridge construction. However, the tensile characteristics and durability predominantly influence the service life of BDLC. While mud powder does contribute to the increased compressive strength of concrete in the later stages, its presence in the aggregates results in reduced tensile properties and diminished durability of BDLC. As a result, it is crucial to minimize the mud powder content in the aggregates, considering the various compositions of mud powder and the specific operational conditions of BDLC in regions subject to seasonal freezing.
(6) No significant correlation was observed between the ITZ and compressive strength or static compressive elastic modulus. However, a clear relationship was established between the properties of the ITZ and tensile characteristics and durability. Further research is required to fully understand the nature and characteristics of the ITZ.

## Figures and Tables

**Table 1:** Chemical composition and physical properties of cement and fly ash.

Chemical Composition (wt.%)	Cement	Fly Ash	Physical Properties	Cement	Fly Ash
${\mathrm{SiO}}_{2}$	21.571	62.219	$\mathrm{Specific}\;\mathrm{surface}\;(m^{2}/\mathrm{kg}$)	340	544
${\mathrm{Al}}_{2}O_{3}$	4.957	20.203	$\mathrm{Density}\;(g/{\mathrm{cm}}^{3}$)	2.9	2.40
${\mathrm{Fe}}_{2}O_{3}$	4.909	2.915	Initial setting time (min)	185	-
CaO	62.094	8.906	Final setting time (min)	250	-
MgO	1.467	0.815	3d Flexural strength (MPa)	6.7	-
${\mathrm{Na}}_{2}O$	0.344	1.357	28d Flexural strength (MPa)	9.4	-
$K_{2}O$	0.499	2.170	3d Compressive strength (MPa)	27.9	-
${\mathrm{SO}}_{3}$	3.417	0.620	28d Compressive strength (MPa)	60.8	-
Ignition loss	2.3	3.1	Activity index (28d)	-	84%

**Table 2:** Properties of river sands and coarse aggregate.

Properties	$\mathrm{Apparent}\;\mathrm{Density}\;(\mathrm{kg}/m^{3}$)	Clay Content (%)	Crush Value (%)	Fineness Modulus
Coarse aggregate	2790	0.60	13.4	-
River sands	2630	1.35	-	2.9

**Table 3:** Properties of polypropylene fiber.

Diameter (μm)	Length (mm)	Elastic Modulus (GPa)	Tensile Strength (MPa)	Elongation (%)
20	6	4.5	530	22

**Table 4:** The aggregates for each test group.

Group	Aggregates
C-0	Washed aggregates
C-SC	Unwashed aggregates
C-C	Washed aggregates, re-incorporation of mud powder sieved from unwashed coarse aggregate with a particle size of less than 75 μm
C-S	Washed aggregates, re-incorporation of mud powder sieved from unwashed river sands with a particle size of less than 75 μm
C-K	Washed aggregates, re-incorporation of clay powder whose main ingredient is kaolin with a particle size of less than 75 μm

**Table 5:** The chemical composition of mud powder.

Chemical Composition (wt.%)	Mud Powder-C	Mud Powder-S	Mud Powder-K
${\mathrm{SiO}}_{2}$	55.514	68.418	50.650
${\mathrm{Al}}_{2}O_{3}$	18.562	16.567	47.511
${\mathrm{Fe}}_{2}O_{3}$	7.304	2.714	0.514
CaO	7.447	2.487	0.154
MgO	4.367	0.798	0.061
${\mathrm{Na}}_{2}O$	2.921	3.971	0.066
$K_{2}O$	2.072	3.953	0.145
${\mathrm{SO}}_{3}$	0.116	0.125	0.024
${\mathrm{TiO}}_{2}$	0.982	0.566	0.793
$P_{2}O_{5}$	0.317	0.199	0.032

Mud powder-C, Mud powder-S, and Mud powder-K represent, respectively, mud powder sieved from unwashed coarse aggregate with a particle size of less than 75 μm, mud powder sieved from unwashed river sands with a particle size of less than 75 μm and clay powder, whose main ingredient is kaolin with a particle size of less than 75 μm.

**Table 6:** Concrete mixture proportions.

Sample	Mixture Proportion/$(\mathrm{kg}/m^{3}$)								
	Cement	Fly Ash	Water	Stone	River Sand	PPF	SP (RAWY-101)	Inhibitor (SBT-TIA)	Clay
C-0	416	74	156	1070	713	0.9	4.165	15	0
C-SC	416	74	156	1060.37	706.583	0.9	5.145	15	16.047
C	416	74	156	1053.953	713	0.9	5.145	15	16.047
C-S	416	74	156	1070	696.953	0.9	4.9	15	16.047
C-K	416	74	156	1060.37	706.583	0.9	5.39	15	16.047

PPF and SP represent polypropylene fiber and polycarboxylate superplasticizer, respectively.

**Table 7:** Results at the end of freeze–thaw cycles in water.

Group	Mass Loss Rate (%)	Relative Dynamic Elastic Modulus (%)	Relative Durability Index (%)	Maximum Number of Freeze–Thaw Cycles
C-0	5.31	71.66	107.49	450
C-SC	5.17	69.89	99.01	425
C-C	5.19	70.61	100.03	425
C-S	5.26	68.94	97.67	425
C-K	5.66	64.43	91.28	425

**Table 8:** Results at the end of freeze–thaw cycles in salt solution.

Group	Mass Loss Rate (%)	Relative Dynamic Elastic Modulus (%)	Relative Durability Index (%)	Maximum Number of Freeze–Thaw Cycles
C-0	4.73	55.57	74.09	200
C-SC	4.02	58.99	68.83	175
C-C	4.03	59.34	69.23	175
C-S	4.08	57.97	67.63	175
C-K	4.24	54.84	63.97	175

## Data Availability

The data presented in this study are available on request from the corresponding author.
